# Interlaboratory validation data on real-time polymerase chain reaction detection for unauthorized genetically modified papaya line PRSV-YK

**DOI:** 10.1016/j.dib.2016.03.095

**Published:** 2016-04-01

**Authors:** Kosuke Nakamura, Kazunari Kondo, Hiroshi Akiyama, Takumi Ishigaki, Akio Noguchi, Hiroshi Katsumata, Kazuto Takasaki, Satoshi Futo, Kozue Sakata, Nozomi Fukuda, Junichi Mano, Kazumi Kitta, Hidenori Tanaka, Ryo Akashi, Tomoko Nishimaki-Mogami

**Affiliations:** a National Institute of Health Sciences, 1-18-1 Kamiyoga, Setagaya-ku, Tokyo 158-8501, Japan; b FASMAC CO., LTD., 5-1-3 Midorigaoka, Atsugi, Kanagawa 243-0041, Japan; c Analytical Science Division, National Food Research Institute, National Agriculture and Food Research Organization, 2-1-12 Kannondai, Tsukuba, Ibaraki 305-8642, Japan; d Graduate School of Agriculture, University of Miyazaki, 1-1 Gakuen Kibanadai Nishi, Miyazaki 889-2192, Japan

**Keywords:** Genetically modified, Real-time PCR, *Carica papaya* L., Validation data

## Abstract

This article is referred to research article entitled “*Whole genome sequence analysis of unidentified genetically modified papaya for development of a specific detection method*” (Nakamura et al., 2016) [1].

Real-time polymerase chain reaction (PCR) detection method for unauthorized genetically modified (GM) papaya (*Carica papaya* L.) line PRSV-YK (PRSV-YK detection method) was developed using whole genome sequence data (DDBJ Sequenced Read Archive under accession No. PRJDB3976). Interlaboratory validation datasets for PRSV-YK detection method were provided. Data indicating homogeneity of samples prepared for interlaboratory validation were included. Specificity and sensitivity test data for PRSV-YK detection method were also provided.

## Specifications table

**Table:** 

Subject area	Chemistry, Biology
More specific subject area	Food analysis
Type of data	Table, figure
How data was acquired	Real-time PCR using ABI PRISM 7900HT Sequence Detection System (Thermo Fisher Scientific Inc.)
Data format	Raw, analyzed
Experimental factors	Purified GM papaya DNA content (0%, 0.05% and 0.10% [w/w]), real-time PCR at 12 laboratories
Experimental features	Interlaboratory validation, specificity and sensitivity testing
Data source location	Chigasaki, Kawasaki, Kobe, Saitama, Tama, Tokyo and Yokohama, Japan
Data accessibility	Data available within this article

## Value of the data

• The data support development of real-time PCR detection method for GM papaya using whole genome sequence data.
• The data provide information on reliability of developed real-time PCR method to detect GM papaya line PRSV-YK.
• The data support developed real-time PCR method use in monitoring foods for GM papaya line PRSV-YK contamination.

## 1 Data

**Fig. 1:**
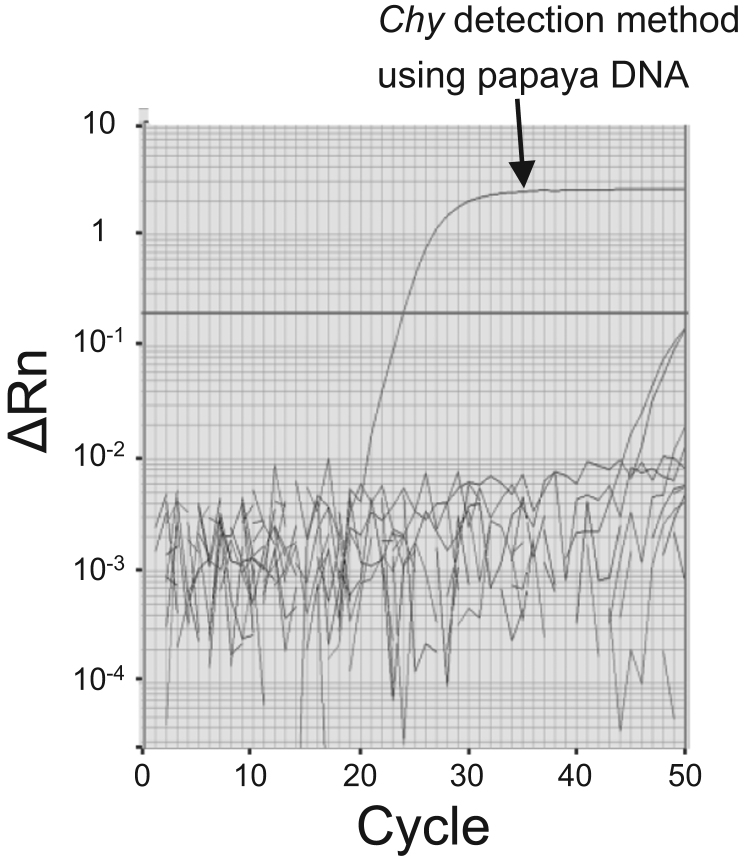
Real-time PCR amplification plot using PRSV-YK and *Chy* detection methods. A duplicate test was done using 50 ng DNA purified from papaya, rice, soybean, maize, potato, rapeseed, pineapple, peach and passion fruit.

**Fig. 2:**
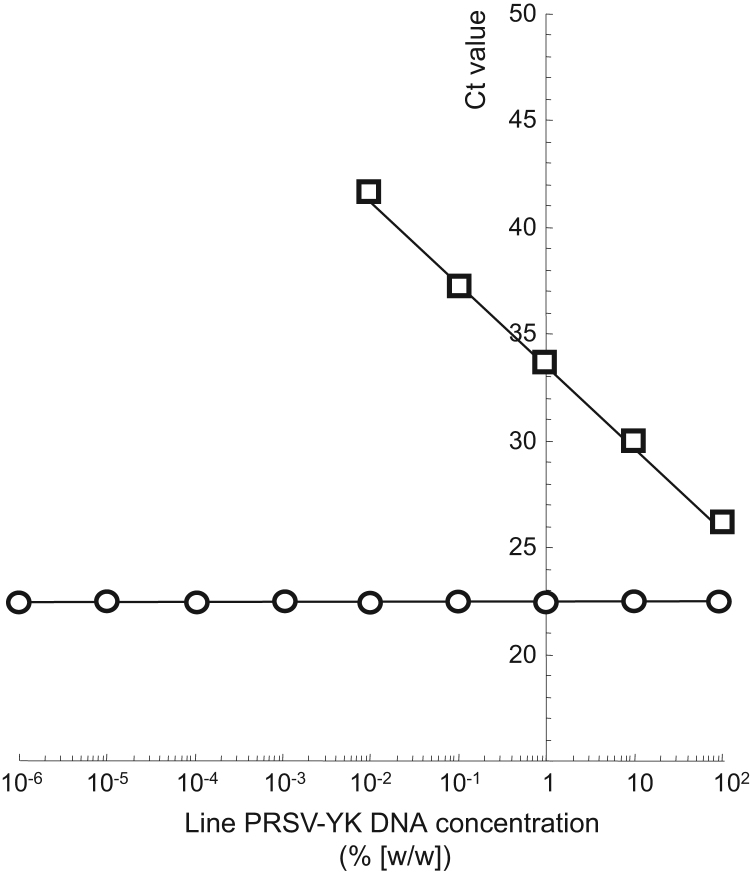
Evaluation of PRSV-YK and *Chy* detection methods. DNA from line PRSV-YK was used to prepare a series of eight-fold serial dilutions (10^−6^–100%) with non-GM papaya DNA. DNA sample (50 ng) was used as a template for real-time PCR. Shown is a representative plot of mean Ct values obtained from duplicate tests at each concentration of DNA sample. PRSV-YK detection method (☐), *Chy* detection method (〇).

Datasets provided in this article represent reliability of unauthorized genetically modified (GM) papaya (*Carica papaya* L.) line PRSV-YK detection method (PRSV-YK detection method), including papaya endogenous gene, *Chymopapain (Chy)*, detection method, using real-time polymerase chain reaction (PCR). Table 1 presents specificity of PRSV-YK and *Chy* detection methods. Fig. 1 shows that *Chy* detection method amplified papaya DNA, but both PRSV-YK and *Chy* detection methods did not amplify rice, soybean, maize, potato, rapeseed, pineapple, peach or passion fruit DNA. Fig. 2 presents sensitivity of PRSV-YK detection method. Cycle threshold (Ct) values obtained from real-time PCR amplification plot were quantitative (*R*^2^=0.99) in the range of 0.01–100% line PRSV-YK DNA concentrations. Table 2 presents results of homogeneity test on prepared samples. Table 3 presents statistical data obtained from homogeneity test on prepared samples. Table 4 summarizes interlaboratory validation data. Data were statistically analyzed to determine mean, relative standard deviation (RSD), repeatability RSD (RSD_r_) and reproducibility RSD (RSD_R_) from Ct values obtained [1].

## 2 Experimental design, materials and methods

### 2.1 Preparation of samples for interlaboratory validation

DNA purified from fresh papaya fruit was used as sample. DNA purified from GM papaya was mixed with DNA from non-GM papaya to prepare a dilution series of GM papaya DNA. Samples were prepared at three different levels of GM papaya DNA concentrations (0%, 0.05% and 0.10% [w/w]). Aliquots of the diluted DNA were placed in individual tubes. Each tube was then labeled with a randomized number. Six randomly selected tubes of each analyte concentration were tested as blind samples at each participating laboratory owning an ABI PRISM 7900HT Sequence Detection System (Thermo Fisher Scientific Inc.). Samples and real-time PCR primer and probe solutions were stored frozen at −20 °C until use.

### 2.2 Confirmation of homogeneity of samples

According to a procedure described by Thompson et al. [2], homogeneity of samples was verified before dispatching them to participating laboratories. Ten test samples of each GM papaya DNA concentration (0%, 0.05% and 0.10% [w/w]) were labeled with a randomized number, and randomly selected samples were used. Each blind sample was tested to determine Ct values at threshold value 0.2 from exponential amplification plots obtained using developed real-time PCR method [1]. Data were analyzed by Cochran׳s test and one-way analysis of variance.

### 2.3 Interlaboratory validation

Method for interlaboratory validation was followed as described previously [2], [3]. Twelve laboratory participants were organized to evaluate repeatability and reproducibility of developed real-time PCR method. Reagents and accessories necessary for real-time PCR and experimental protocol were provided to each participating laboratory. ABI PRISM 7900HT Sequence Detection System, owned by each lab, was used for analyses. Real-time PCR was conducted within three months. All data were collected from 12 laboratories. Presence of line PRSV-YK in samples was judged by Ct values at threshold value 0.2 (present, Ct<48.00; absent, Ct≥48.00) obtained using PRSV-YK and *Chy* detection methods. To statistically analyze interlaboratory validation data, Ct values from all laboratories were used after eliminating outliers by a 1-tailed Cochran׳s test at a probability value of 2.5%.

## Figures and Tables

**Table 1:** Specificity data on developed detection method.^a^

Sample	Detection method^b^	
*Chy*	PRSV-YK
Papaya	23.93/23.91	−/−
Rice	−/−	−/−
Soybean	−/−	−/−
Maize	−/−	−/−
Potato	−/−	−/−
Rapeseed	−/−	−/−
Pineapple	−/−	−/−
Peach	−/−	−/−
Passion fruit	−/−	−/−

a Ct values (threshold value at 0.2) were recorded from duplicate test using DNA purified from each sample.

b −, scored negative.

**Table 2:** Results of homogeneity test on prepared samples.

Line PRSV-YK DNA concentration (w/w [%])	Detection method				Result	
PRSV-YK (30)^a^		*Chy* (30)^a^	
(+)^b^	(−)^b^	(+)^b^	(−)^b^	Positive	Negative
0	0	10	10	0	0	10
0.05	10	0	10	0	10	0
0.10	10	0	10	0	10	0
						
Expected	20	10	30	0	20	10
Agreement^c^ (%)	100	100	100	100	100	100

a The number in brackets indicates the total number of samples analyzed.

b (+) = number of positive reactions, (−) = number of negative reactions.

c The value was calculated from comparison of the expected results with the results obtained from homogeneity test.

**Table 3:** Statistical data obtained from homogeneity test on prepared samples.

Line PRSV-YK DNA concentration (w/w [%])	*n*	ΔCt_PRSV-YK – *Chy*_^a^	RSD^b^ (%)	*F*-ratio	*F* crit^c^	Result (*F*-ratio<*F* crit)
0.05	10	15.54	3.24	1.59	3.02	Acceptable
0.10	10	14.29	1.97	2.47	3.02	Acceptable

a Difference in Ct values (at threshold value 0.2) obtained using PRSV-YK and *Chy* detection methods.

b RSD%, calculated from Sa (SD of analysis).

c *F* crit, critical *F* value.

**Table 4:** Interlaboratory validation data.

Laboratory	Detection method										Result		
PRSV-YK (36)^a^					*Chy* (36)^a^				
(+)^b^	Line PRSV-YK DNA concentration (w/w [%])	Mean	RSD (%)	(−)^b^	(+)^b^	Line PRSV-YK DNA concentration (w/w [%])	Mean	RSD (%)	(−)^b^	Positive	Negative	
A	24	0.05	38.09	2.33	12	36	0.05	22.62	0.44	0	12		6
		0.10	37.52	1.07			0.10	22.57	0.41				
B	24	0.05	38.22	1.35	12	36	0.05	22.76	0.30	0	12		6
		0.10	37.31	1.11			0.10	22.74	0.29				
C	24	0.05	38.44	1.29	12	36	0.05	22.60	0.64	0	12		6
		0.10	37.37	1.69			0.10	22.60	0.70				
D	24	0.05	38.54	1.21	12	36	0.05	22.91	0.54	0	12		6
		0.10	37.55	1.64			0.10	22.85	0.29				
E	24	0.05	38.17	1.38	12	36	0.05	22.71	0.55	0	12		6
		0.10	37.07	0.95			0.10	22.74	0.54				
F	24	0.05	38.13	1.48	12	36	0.05	22.46	0.43	0	12		6
		0.10	37.51	1.32			0.10	22.46	0.34				
G	24	0.05	38.91	1.97	12	36	0.05	22.36	0.14	0	12		6
		0.10	37.97	2.01			0.10	22.38	0.35				
H	24	0.05	38.15	0.81	12	36	0.05	22.37	0.45	0	12		6
		0.10	37.27	1.41			0.10	22.43	0.36				
I	24	0.05	38.51	1.37	12	36	0.05	22.73	0.36	0	12		6
		0.10	37.52	1.78			0.10	22.75	0.30				
J	24	0.05	38.37	1.41	12	36	0.05	22.65	0.26	0	12		6
		0.10	37.87	0.94			0.10	22.57	0.31				
K	24	0.05	38.31	1.32	12	36	0.05	22.65	0.32	0	12		6
		0.10	37.11	0.93			0.10	22.55	0.49				
L	24	0.05	37.70	1.25	12	36	0.05	22.27	0.19	0	12		6
		0.10	36.56	0.95			0.10	22.30	0.66				
Expected	24				12	36				0	12		6
Agreement^c^ (%)	100				100	100				100	100		100

a The number in brackets indicates total number of samples analyzed per a laboratory.

b (+) = number of positive reactions, (−) = number of negative reactions.

c The value was calculated from comparison of the expected results with the results obtained from interlaboratory validation.
