# Supplementary material for: Interlaboratory validation data on real-time polymerase chain reaction detection for unauthorized genetically modified papaya line PRSV-YK
Source: Data Brief. 2016 Apr 1;7:1165–70. doi: 10.1016/j.dib.2016.03.095 (PMC4927967; doi:10.1016/j.dib.2016.03.095)
Supplement: Supplementary file 1 — Supplementary material [file mmc1.pdf]

現在の PDF ビューアはこのファイルの内容を表示できません。最新のバージョンにアップデートしてください。
